# Weakly Supervised 2D Pose Adaptation and Body Part Segmentation for Concealed Object Detection †

**DOI:** 10.3390/s23042005

**Published:** 2023-02-10

**Authors:** Lawrence Amadi, Gady Agam

**Affiliations:** Visual Computing Lab, Illinois Institute of Technology, Chicago, IL 60616, USA

**Keywords:** pose refinement, 2D pose correction, domain adaptation, body segmentation, body part recognition, object detection, anomaly detection, threat localization

## Abstract

Weakly supervised pose estimation can be used to assist unsupervised body part segmentation and concealed item detection. The accuracy of pose estimation is essential for precise body part segmentation and accurate concealed item detection. In this paper, we show how poses obtained from an RGB pretrained 2D pose detector can be modified for the backscatter image domain. The 2D poses are refined using RANSAC bundle adjustment to minimize the projection loss in 3D. Furthermore, we show how 2D poses can be optimized using a newly proposed 3D-to-2D pose correction network weakly supervised with pose prior regularizers and multi-view pose and posture consistency losses. The optimized 2D poses are used to segment human body parts. We then train a body-part-aware anomaly detection network to detect foreign (concealed threat) objects on segmented body parts. Our work is applied to the TSA passenger screening dataset containing millimeter wave scan images of airport travelers annotated with only binary labels that indicate whether a foreign object is concealed on a body part. Our proposed approach significantly improves the detection accuracy of TSA 2D backscatter images in existing works with a state-of-the-art performance of 97% F1-score, 0.0559 log-loss on the TSA-PSD test-set, and a 74% reduction in 2D pose error.

## 1. Introduction

Backscatter images such as X-ray, MRI, and millimeter wave scan images are predominantly used to examine internal body organs or to screen beneath the clothing of people. These images are generated by specialized scanners and are essential for computer-aided medical diagnosis and security screening inspections. Backscatter images are also characterized by very low chromaticity and illumination. The poor visibility makes human inspection difficult and also poses a challenge for computer diagnostic software. In the United States, the HIPAA Privacy Rule considers these intrusive backscatter images as the personal medical data of the human subject and protects it against unauthorized inspection. Hence, computer vision algorithms are used to analyze and detect anomalies in airport security screenings where full-body backscatter millimeter wave scan (MWS) images of people must be inspected for concealed, prohibited, or harmful items stowed underneath garments. We refer to such items as *threat objects*. When a threat object is detected, the algorithm must also localize it to a specific body part as it recommends a follow-up pat-down search of the indicated body part. Therefore, localizing anomalies to specific body parts is as important as high-precision anomaly detection of concealed items to streamline pat-down searches by security personnel and shorten airport security screening queues. This requirement demands an algorithm capable of recognizing different body parts in backscatter MWS images. Similarly, for medical imaging, there is value in developing an anomaly detection algorithm that is organ and body part aware to assist physicians in diagnosis. In this work, we propose an unsupervised body part segmentation approach that enables us to train a body-part-aware anomaly detection CNN to detect concealed threat objects on segmented body parts.

**Figure 1:**
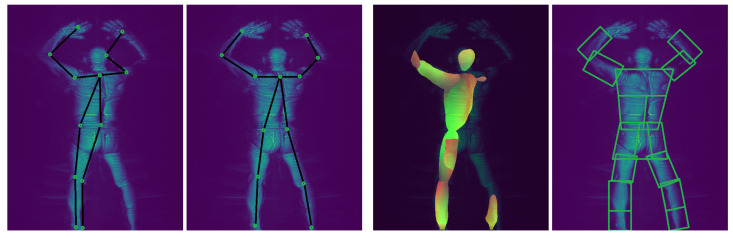
Example of our refined 2D pose (2nd) and unsupervised body segmentation (4th) on MWS images compared to SOTA HRNet 2D pose (1st) [2] and DensePose body segmentation (3rd) [1].

Designing a body-aware anomaly detection algorithm for backscatter images is challenging for two reasons. First, unlike RGB images, there are hardly datasets of backscatter MWS images with body part bounding-box or pixel-level annotations sufficiently large enough to supervise the training of a body part segmentation neural network for backscatter images. Second, as demonstrated in Figure 1, directly applying pretrained RGB body segmentation models (e.g., DensePose [1]) to MWS images fails to produce meaningful segmentation because of inadequate chromatic and illumination cues.

Therefore, this work makes the following contributions:

1. We propose two methods for adapting an RGB pretrained pose detector to estimate accurate 2D poses in backscatter images, including a weakly supervised 3D–2D pose correction network that is optimized without ground-truth (GT) 2D or 3D pose annotations. Our proposed 2D pose refinement significantly improves the correctness of 2D poses in backscatter images.
2. We introduce an unsupervised procedure for segmenting human body parts in MWS images by estimating bounding polygons for each body part using keypoints of refined 2D poses. Our heuristic approach is motivated by the absence of body part annotations in the dataset and is effective at segmenting all body parts of a person in images captured from different viewpoints.
3. We then propose a weakly supervised, ROI-attentive, dual-chain CNN classifier that detects anomalies given multi-view images of a cropped body part. Our anomaly detection network processes segmented images, their region of interest (ROI) masks, and derived region composite vectors (RCV) to decide if a detected anomaly should be attributed to a given body part. This ensures the network not only detects anomalies but also learns to associate anomalies with affected body parts, ultimately leading to more precise detection.

Our proposed approach leverages multi-view information to refine sub-optimal poses generated by RGB pretrained human pose estimators. The refined keypoints are then used to estimate bounding polygons that enclose each body part. Subsequently, the bounding polygons are used to crop regions of the images that represent each body part and the images are fed to our body-aware anomaly detection neural network for inspection.

In a preceding conference paper [3], we showed how 2D pose estimation can be used to perform unsupervised body part segmentation and concealed object detection. In this paper, we expound on the conference paper, adding more evaluation results and details about our methods. In addition, we introduce a novel approach to improve estimated 2D poses using a weakly supervised 3D-to-2D pose correction network. We propose a weakly supervised pose correction network that is trained by optimizing multi-view pose and posture consistency loss terms [4] and biomechanical pose prior regularizers [5].

### TSA Passenger Screening Dataset

**Figure 2:**
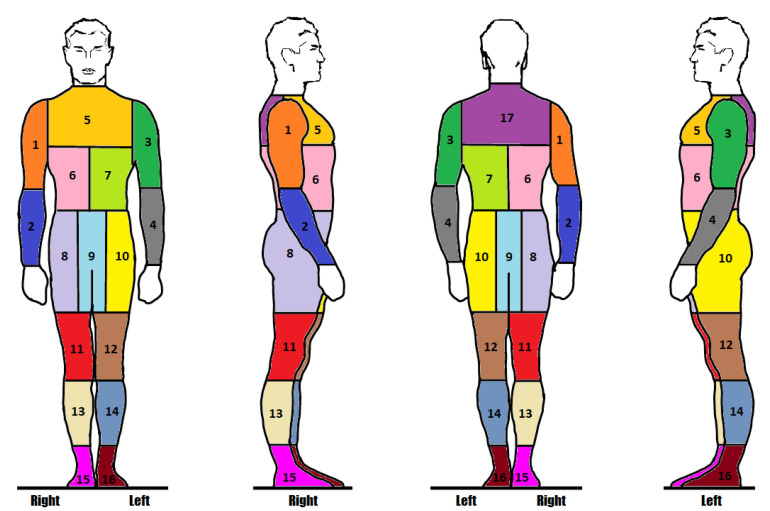
The 17 body zones of interest as indicated by the TSA Passenger Screening Dataset.

**Figure 3:**
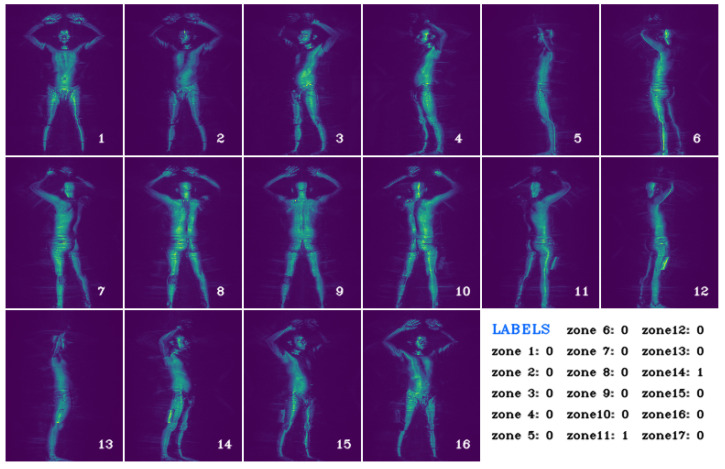
An example *.aps* scan with 16 frames and corresponding binary GT labels for the 17 body zones that indicate the presence of a concealed item in the zone or body part. Notice the threat objects on the right lower thigh (zone 11) and left calf (zone 14) visible in frames 10–16 and 7–10, respectively.

Our proposed unsupervised body segmentation and concealed object detection method is evaluated on the Transportation Security Administration Passenger Screening Dataset (TSA-PSD) [6] which contains backscatter full-body scans of people acquired by a High Definition Advanced Imaging Technology (HD-AIT) millimeter wave scanner (MWS). The dataset contains 2635 scans of airport travelers. A total of 1388 of the 2635 scans are the reserved test set with private annotations known only to TSA. The remaining 1247 scans are the annotated training set with binary labels that indicate whether an object is concealed within a body part of the scan subject. TSA outlines 17 body parts of interest illustrated in Figure 2. They include right and left forearms (2 and 4), biceps (1 and 3), abdomens (6 and 7), upper and lower thighs (8, 10, 11, and 12), calves (13 and 14), ankles (15 and 16), upper back (17), chest (5), and groin (9). There are no pixel-level or image-level ground-truth annotations of concealed items or body parts. The provided binary labels indicate if a threat object is concealed on a body part given a scan. It does not indicate in which frames the concealed item is visible. Figure 3 depicts an example of the frames (16) and corresponding 17 zones’ ground-truth labels of a scan.

**Figure 4:**
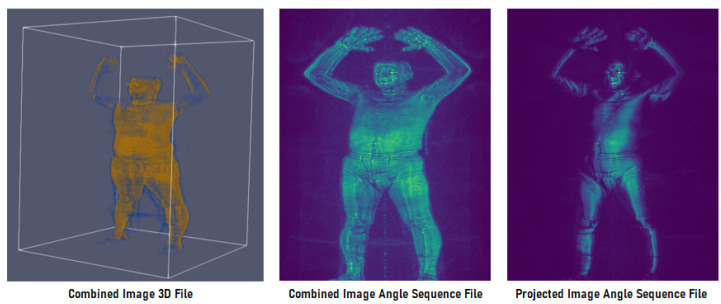
From left to right. Examples of image renderings of *.a3d*, *.a3daps* (frame 4 of 64), and *.aps* (frame 2 of 16) TSA dataset file formats of the same subject.

TSA provides four data formats for each scan captured by the HD-AIT millimeter wave scanner. Starting from the largest to the smallest data format, the **Calibrated Object Raw Data File (2.26 GB per file,** ***.ahi*** **file extension)** is the raw data file produced by the HD-AIT millimeter wave scanner from which the following three data formats are generated. The **Combined Image 3D File (330 MB per file,** ***.a3d*** **file extension)** is a collection of eight 3D images that are equally spaced around the region scanned and combined into one composite 3D volumetric image. When played back plane-by-plane, this image displays cross-section slices of the person at sequential heights. The **Combined Image Angle Sequence File (41.2 MB per file,** ***.a3daps*** **file extension)** is a 2D projection of the 3D data at sequential angular increments that are written contiguously into a single file. The rendering of each file produces 64 2D images that, when played back frame-by-frame, show the subject spinning from left to right on the screen. The **Projected Image Angle Sequence File (10.3 MB per file,** ***.aps*** **file extension)** maps the 3D backscatter image of a scan to 90-degree segments of data that are equally spaced around the region scanned. A maximum value projection of each mapping is written sequentially into a single file from which 2D images are generated. Similar to *.a3daps* files, this results in a sequence of 16 2D images captured from different viewpoints such that the person appears to be spinning from left to right when the images are played back frame-by-frame. Figure 4 shows examples of the latter three data formats.

In this work, we exclusively work with the 2D image (*.aps* and *.a3daps*) file formats for two reasons. First, 2D images are more common than 3D images. This popularity means that we can take advantage of existing computer vision methods and neural networks proposed for 2D images. It also means that the methods we develop in this work are applicable to other types of 2D backscatter images. Second, the 2D image renderings are derived from smaller-sized files and therefore require less memory resources to analyze. This allows us to design memory-efficient and lightweight algorithms in comparison to using larger 3D files. The major drawback to using 2D image formats, however, is the loss of information that may be critical to detection when 3D data are downsampled to 2D. We focus our efforts on the lightest Projected Image Angle Sequence File. Hence, we retrieve 16 images for each scan with 17 binary labels that indicate the presence of a concealed item in each of the 17 body parts. This dataset poses a difficult challenge because of the minimal ground-truth labels provided for the task of body-part anomaly detection and localization. We do not have pixel-level or bounding-box annotations of body parts or concealed items. This means that we cannot train a supervised body part segmentation network. We also cannot assume that a concealed item, when present, is visible from all viewpoints. Hence, we cannot detect anomalies on a per-image basis. Rather, concealed items on a body part must be detected on a per-image-sequence basis. Therefore, we propose a method for unsupervised body part recognition and segmentation followed by a minimally supervised anomaly detection on segmented body parts.

## 2. Related Work

We discuss existing works in various categories related to the task of concealed object detection and localization to human body parts.

**Salient Object Detection.** Over the years, various autoencoder-based salient object detection and segmentation methods have been proposed. However, most of the methods are either optimized for one salient object per image or multiple objects in a scene, whereas the scope of this body part anomaly detection task is a case of multi-object detection on multiple images. Moreover, the salient object separation techniques proposed in [7,8,9,10,11,12] do not perform well on MWS images which are characterized by low pixel intensity and lacking chromatic information. The presence of ghosting artifacts, common in backscatter imaging, adds pixel-level noise which makes salient object detection even more difficult. Mask-RCNN [13] is one such state-of-the-art deep network architecture designed for object detection and pixel-level localization in RGB images. It is a multi-label network that detects whether or not certain objects are present in an image while simultaneously masking the pixel regions where the objects are located. Mask-RCNN is, however, designed for single-image inputs. Although, in theory, the architecture can be extended to process multiple images per sample. Mask-RCNN requires pixel-level annotations of objects of interest to train the network. However, the TSA dataset lacks such annotations. Moreover, pixel-level localization of objects is not enough. In the case of body part anomaly detection, the model must map highlighted pixels to a specific human body part. Hence, body part recognition is important and must be addressed in the scope of this work.

**Anomaly Object Detection.** This has been studied in people, luggage, cargo containers, and scenes in [8,14,15,16,17,18,19]. The majority of the leading methods are based on deep neural networks. Riffo and Mery [15] proposed a shape implicit algorithm for detecting specific threat objects (razor blades, shurikens, and guns) in X-ray images of luggage. Although their method can be modified to detect threat items on the human body, their object-specialized approach is not expected to generalize to unknown objects that are not encountered during training because their algorithm is designed to detect specific items, not general anomalies. A popular approach for detecting threat objects uses the AlexNet classifier [18] and predefined fixed region of interest (ROI) bounding boxes to segment body parts. The fixed ROI bounding boxes do not account for variations in body part size, positioning, and orientation on a per-person basis. This limitation makes this approach more suitable for the less mobile torso body parts (e.g., chest and back) and limited viewpoints. Another approach that uses AlexNet for anomaly detection [19] combines 2D and 3D data to segment the body parts and generate threat or benign labels for each cropped image. This enables supervised training of the model with a set of cropped body parts and assigned threat labels. This approach allows for a simpler neural network architecture because of the one-to-one mapping of cropped images and labels. However, generating false labels for cropped images can degrade the accuracy of the classifier. Note, anomalies in TSA-PSD body parts are typically visible in six frames or less.

**Concealed Object Detection on TSA-PSD.** Other concealed item detection algorithms applied to TSA-PSD are either designed to use 3D volumetric data or a combination of 3D and 2D images. We give a high-level description of the proprietary TSA-PSD classifiers as reported by the Department of Homeland Security, as details of the state-of-the-art classifiers are not released to the public. Jeremy Walther’s (1st) approach used an array of deep learning models customized to process images from multiple views. Sergei Fotin (2nd) and Oleg Trott (5th) adopted an approach that fuses 2D (10–41 MB per file) and 3D (330 MB per file) data sources to make object and location predictions. Despite their high accuracy, the 1st and 2nd methods may be less suitable for real-time use because the inference time for an array of neural networks or very large files can be substantial. David Odaibo and Thomas Anthony (3rd) developed an algorithm that uses specialized 3D image-level annotations to train a two-stage identification model. It is unclear whether the annotations were automated or manually labeled. Location-based models (4th), automatic image segmentation with a collection of specialized models trained on cropped body part images (6th), separately trained models with image augmentation (8th), and the use of synthetic data and cross-image analysis (7th) are other techniques used to improve model detection accuracy.

Our approach to body part anomaly detection is to split the task into body part segmentation and anomaly detection on segmented body parts [3]. We propose a body part segmentation method that extends 2D human pose estimation. The 2D pose estimation on RGB images is widely studied in [2,20,21,22,23,24]. However, these models require keypoint annotations to learn accurate pose encoding. Similarly, state-of-the-art human body segmentation neural networks [1,13,25,26,27,28,29,30,31,32,33,34,35] rely on bounding-box or pixel-level annotations for supervised body part segmentation. Given the absence of such annotations in the TSA datasets, we cannot directly use these supervised models. Instead, we propose two methods (unsupervised and weakly supervised) for adapting an RGB pretrained 2D pose detector to estimate accurate poses in MWS images. We then propose an unsupervised method for segmenting human body parts given refined (or corrected) 2D keypoints. Finally, we propose a region-attentive anomaly detection network for detecting concealed items on cropped images of segmented body parts.

## 3. Method

We approached the task of concealed item detection and localization to human body parts as a two-stage problem. First, we segmented the human body parts in the frames of each scan to generate 17 sequences of n≤16 cropped images (Section 3.1). Each sequence corresponds to a body part and contains cropped images of that body part from different viewpoints. Since the presence of a concealed item is often visible in six frames or less, never from all viewpoints, we must detect anomalies in body parts on a per image-sequence basis. In the second stage (Section 3.2), we trained a deep CNN anomaly detector that processes a sequence of cropped images of a body part and classifies it as benign or abnormal when a foreign object is detected in any of the cropped images. Note, a single CNN detector is trained for all body parts. This makes our detector simpler and lightweight compared to other state-of-the-art classifiers that use an array of body parts or gender-specialized networks. In contrast, our network uses a novel region-attentive architecture that makes it aware of the different body parts.

### 3.1. Unsupervised Body Part Segmentation from 2D Poses

Our approach to unsupervised body part segmentation relies on accurately estimated 2D pose keypoints. We later describe a heuristic method that uses the keypoints in each frame as landmarks to identify and outline the human’s body parts (Section 3.1). We have demonstrated in Figure 1 that 2D pose detectors trained on RGB images do not generalize well to MWS backscatter images and often estimate inaccurate poses. We, therefore, propose two methods for adapting the output of RGB trained 2D *Human Pose Estimation (HPE)* networks to MWS images. (1) Our first 2D pose correction approach begins with an informed selection of local optima keypoint locations from the confidence map outputs of an RGB pretrained 2D pose estimator by leveraging certain domain knowledge of the TSA dataset (Section 3.1.1). The corrected keypoint positions in each frame are further optimized using RANSAC bundle adjustment [36] to consolidate a global optimum 3D pose. A new set of coherent 2D poses are derived by projecting the global optimum 3D pose back to the 2D frames (Section 3.1.2). (2) Our second approach for correcting sub-optimal estimated 2D keypoints uses a weakly supervised 3D pose estimator to encode an optimal 3D pose for each frame. We then project the 3D pose back to 2D to retrieve the reprojected and corrected 2D pose for the frame (Section 3.3). This new approach is the extension of the original RaadNet paper [3]. Both approaches (RANSAC bundle adjustment and learned pose correction) involve up-sampling to a higher (3D) dimension where more reliable constraints (multi-view consistency and pose geometry) can be enforced and optimized before returning to the lower (2D) dimension to recover the corrected 2D pose. The refined keypoints (from either approach) of the scan subject in a frame are then used to estimate bounding polygons that segment the body parts of the person (Section 3.1.3).

#### 3.1.1. Keypoint Selection from HPE Confidence Maps

Without keypoint annotations to train a pose estimator on the TSA dataset, we use a Deep-HRNet 2D pose estimator [2] pretrained on the COCO dataset [37] to estimate 15 keypoints of people in MWS images. They include right and left wrists, elbows, shoulders, hips, knees, ankles, nose, neck, and pelvis keypoints. Note, the pretrained Deep-HRNet pose detector estimates 17 keypoints. We disregard the eyes’ and ears’ keypoints as they are not needed to segment the body parts of interest. The remaining 13 keypoints are the nose, left and right shoulders, elbows, wrists, hips, knees, and ankles. We then derive the neck and pelvis keypoints by computing the midpoint between the left and right shoulders and hips, respectively. Deep-HRNet is preferred to other 2D HPE networks [20,22,23] because of its high resolution architecture. Compared to the others, it estimates more realistic poses of the subjects in backscatter MWS images. In our initial experiments, we selected the keypoint position by computing the Argmax of the confidence map. This is consistent with the approach in previous 2D pose estimation works as it selects the leftmost pixel location with the highest confidence score. However, we observed that this naive method (referred to as *Generic Pose*) often produced incorrect keypoint estimates because the Deep-HRNet estimator frequently generated confidence maps with more than one concentration of high confidence scores (referred to as blobs) for backscatter MWS images. In such cases, the naive selection will default to the leftmost blob even though the correct keypoint position may be in one of the other blobs.

**Figure 5:**
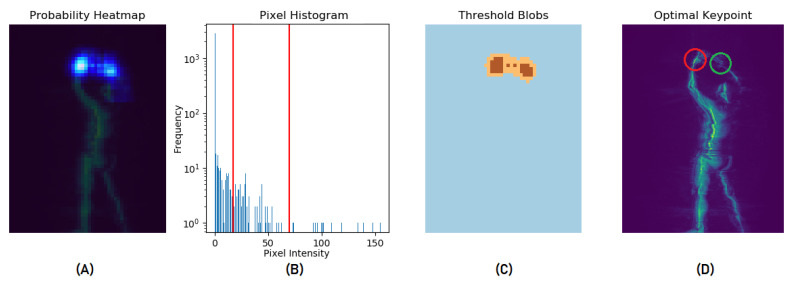
Our keypoint selection algorithm applied to the right wrist. (**A**) depicts the right wrist confidence map. (**B**) is the histogram of the confidence map and the *red* lines show the multi-Otsu thresholds used to segment (**A**) to *blue*, *orange* and *brown* layers in (**C**). (**D**) shows our algorithm selects the correct right wrist position (*green* circle) instead of the most confident location (*red* circle).

We implement a keypoint selection post-processing procedure that selects the best-positioned blob and keypoint location given the occurrence of multiple blobs in the confidence map. The premise of our keypoint selection algorithm is that the relative positioning (left, center, or right) of joints in each frame is consistent across all scans because subjects assume a standard posture (standing erect with hands raised) when being scanned and their pose is sustained while each frame is captured from rotating viewpoints. We begin by segmenting the confidence map into three layers using multi-Otsu binarization [38] to determine the confidence threshold for each layer. Using a modified *island-finder* algorithm, we traverse the segmented confidence map (now a 2D matrix with three unique values; 0, 1, and 2) to identify all blobs. The blobs are grouped into three (or fewer) clusters by spatial proximity. The cluster nearest to the expected keypoint position (left, center, or right) is selected. We then compute the argmax of confidence scores in the chosen blob cluster to retrieve a more appropriate pixel location of the keypoint. Figure 5 illustrates the outcome of this procedure. We refer to the keypoints selected by this procedure as *Confined Pose*.

#### 3.1.2. Multi-View Coherent Pose Optimization

We observed that even after refining the 2D pose in each MWS frame, some keypoints may still be sub-optimally estimated in some frames, subsequently causing inaccurate segmentation of body parts associated with the keypoint. Therefore, we ought to correct incoherent poses across all frames of a scan. In Algorithm 1, we describe a pose optimization procedure that takes advantage of the multiple viewpoints of TSA-PSD MWS images to reconstruct consistent 2D poses across all frames. The 2D position of each keypoint, across all frames of a scan, is optimized (independently of other keypoints) using the RANSAC bundle adjustment algorithm we now discuss [36,39,40,41,42]. The outcome is 2D poses in each frame that are coherent (i.e., the 2D poses in each viewpoint are consistent with a global 3D pose). We refer to the 2D poses generated by the RANSAC bundle adjustment algorithm as *RANSAC-optimized* coherent poses.
**Algorithm 1** Per Keypoint RANSAC Bundle Adjustment**Input:**  $P\leftarrow \{\left(x,y\right):\forall \;frames\;f_{1}\cdots f_{16}\}$▹ 2D pixel positions of keypoint in each frame**Output:**  $P^{"}$▹ 2D pixel positions of keypoint in each frame after bundle adjustment1:  n←0, I←∅
2:  **while** n≤100 **do**▹ iterate over subset of keypoints for 3D bundle adjustment3:      R←{(x,y):⊂P}▹ randomly selected subset, 1 every 4 consecutive frames4:      $p^{3D}\leftarrow {\left(x,y,z\right)}_{R}$▹ 3D point is regressed from *R* via least squares optimization5:      $P^{{}^{\prime }}\leftarrow \left\{\left(x^{{}^{\prime }},y^{{}^{\prime }}\right):\forall \;frames\right\}$▹ 2D positions after projecting $p^{3D}$ to each frame6:      $I^{{}^{\prime }}\leftarrow \left\{\left(x,y\right):\subset P\right\}$▹ note inlier points based on Euclidean dist. between *P* & $P^{{}^{\prime }}$7:      **if** $|I^{{}^{\prime }}\left|>|I\right|$ **then**
8:          $I\leftarrow I^{{}^{\prime }}$▹ retain the largest inlier set9:      **end if**
10:  **end while**
11:  $p^{3D}\leftarrow {\left(x,y,z\right)}_{I}$▹ least squares bundle adjusted 3D point regressed from *I* 2D points12:  $P^{"}\leftarrow \left\{\left(x^{"},y^{"}\right):\forall \;frames\right\}$▹ final 2D positions after projecting $p^{3D}$ to each frame

#### 3.1.3. Estimating Bounding Polygons for Body Segmentation

After refining the keypoints, we segmented the body parts in each frame by estimating a bounding polygon around each body part. Vertices of the **bounding polygon** (a quadrilateral with four vertices) were estimated from a subset of keypoints associated with a given body part. We define two types of body parts. ***Limb Body Parts*** are body parts that can be segmented using a pair of keypoints. They include forearms, biceps, upper and lower thighs, calves, and ankles. We refer to the pair of keypoints used to segment limb body parts as *Anchor keypoints*. ***Torso Body Parts*** are body parts that are segmented using a set of four keypoints. They include the chest, back, abs, and groin. We refer to the keypoints used to segment torso body parts as *Pillar keypoints*.

##### Limb Body Part Segmentation

**Figure 6:**
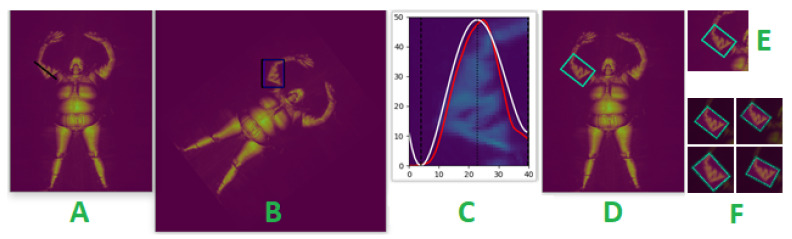
Visualization of intermediate stages of limb segmentation for the right elbow. The black line in (**A**) links the right elbow and shoulder keypoints. The frame is rotated to vertically align the pillar keypoints in (**B**). (**C**) shows the computed *pixel intensity curve* (red line) and fitted polynomial (white) line. (**D**) shows the estimated bounding polygon. (**F**) are examples of (shifted, zoomed, and rotated) cropped image augmentation (overlaid with the ROI mask) generated from (**E**).

We began by computing the angle between the *anchor keypoints* and the y-axis. The image was then rotated by the computed angle so that the limb is vertically aligned. We extracted the luminance channel of the rotated image and removed noise with a Gaussian filter. We then summed the pixel intensities along the horizontal axis of a rectangular region enclosing the limb. This produced a *pixel intensity curve*. The rectangular region is vertically bounded by the y-coordinates of the rotated keypoints and horizontally bounded by a predefined width for each limb. Next, we fitted a six-degree polynomial line to the computed pixel intensity curve and extracted the x-coordinates of the rightmost and leftmost local minima of the polynomial line. The x-coordinates of the local minima define the width of an axis-aligned bounding box around the limb. Similarly, the y-coordinates of the rotated keypoints define the height. The bounding box is transformed into a bounding polygon when its vertices are inversely rotated. This procedure is demonstrated in Figure 6.

##### Torso Body Part Segmentation

**Figure 7:**
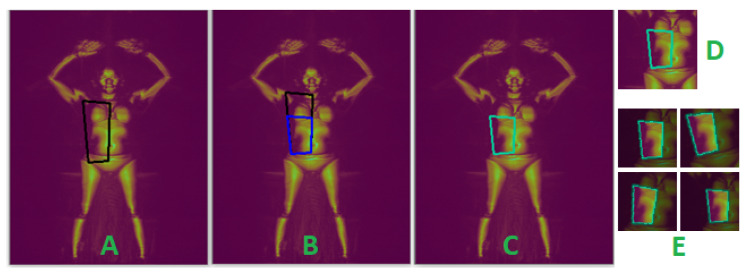
Visualization of torso segmentation for the right abdomen. Vertices of the black quadrilateral in (**A**) are the pelvis, neck, right shoulder, and hip keypoints. The top edge of the black quadrilateral in (**B**) is shifted downwards, resulting in the blue polygon. The green polygon in (**C**) captures the ROI of the right abdomen. (**E**) shows examples of (shifted, zoomed, and rotated) randomly generated cropped image augmentation from (**D**), overlaid with the ROI mask.

The pillar keypoints of torso body parts typically outline a quadrilateral region containing two or more body parts. For example, the right shoulder, neck, pelvis, and right hip keypoints segment the right abdomen and half of the upper chest (see image A in Figure 7). We shift one or more edges of the quadrilateral to precisely capture the intended body part. The edges that are moved, the direction (horizontally or vertical), and the extent they are moved are guided by a predefined configuration for each torso body part. The new vertices of the bounding polygon are computed as the points of intersection of the adjusted quadrilateral edges. This procedure is illustrated in Figure 7 for the right abdomen.

Each bounding polygon defines the region of interest (ROI) of a body part. The segmented body parts are cropped, in excess, by a standard 160×160 pixel window such that the ROI is contained in the cropped image (see E and F in Figure 6 and Figure 7), before downsampling to 80×80 pixels. This approach preserves the aspect ratio of the body parts, in contrast to directly resizing the ROI to the standard size. We found our network performed better at detecting concealed items when the aspect ratios of images were not altered. We have chosen a generous standard size of 160 sq. pixels to accommodate the sizes of all body parts and all subjects, big and small. Another reason for excess cropping of the ROI is that the demarcation between neighboring body parts is not absolute, and when a concealed object spans the boundary of two body parts, it must only be attributed to one. By over-cropping the ROI and incorporating the ROI mask and our proposed region composite vectors into the network, our model learns to associate concealed objects with the dominant body part. We have designed our network architecture to use the ROI mask to refocus attention on the ROI when detecting anomalies, but only after extracting features from the entire cropped images.

To summarize, given each scan, we compiled 17 sequences of cropped images. Each sequence of cropped images corresponds to a body part and the cropped images are the segmented body part from multiple viewpoints. Each sequence contains 12 images because we observed that each body part is visible in at most 12 frames. Cropped images were resampled and augmented to compensate for body parts that are visible in less than 12 frames (as low as three frames for the chest and back). The cropped images are downsized to a tolerable resolution that is appropriate for the anomaly detection network.

### 3.2. RaadNet: ROI Attentive Anomaly Detection Network

**Figure 8:**
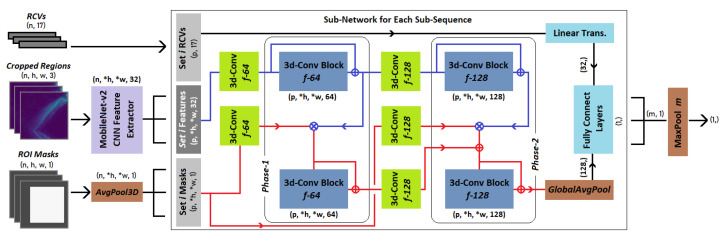
An instance of RaadNet, our two-phase, dual pipeline *(indicated by the blue and red lines)* anomaly detection network that takes as input cropped images of a body part, their ROI masks, and RCVs, and outputs the probability that a concealed item is in either of the cropped images. We use *n* = 12 cropped images per body part. *h* and *w* = 80 are the height and width of the cropped images. ${}^{*}h$, ${}^{*}w$ = 10, *p* = 3, *m* = 4. Each sub-sequence of images *p* is passed through the same sub-network (enclosed in the large rectangle). Residual CNN blocks (in dark blue) contain two 3D convolution layers with *kernel* = 3 and *f* filters. Convolutions are accompanied by batch normalization and Re-LU activation. The fully connected (FCN) block (in light blue) contains five dense layers of sizes 128, 64, 64, 16, and 1 followed by a sigmoid activation. ⊗ and ⊕ are element-wise multiplication and addition operations. Notice that a deeper network can be created by increasing the number of phases.

We designed an anomaly object detection convolution neural network that classifies a sequence of cropped images of a segmented body part as benign or abnormal, indicating the presence of a concealed item in one or more of the cropped images. The network, illustrated in Figure 8, takes an input sequence of *n* = 12 cropped images for each body part, their corresponding ROI binary masks, and region composite vectors (***RCVs***). The RCV of a cropped image is a vector of size 17 defined in Equation (1) as the *intersect-over-union* (*IoU*) between the body part’s ROI, $I_{i}$, and the ROI of all body parts in the given frame, *I*.

(1) $$RCV_{i}\left(I\right)=\langle IoU\left(I_{0},I_{i}\right),\;\cdots ,\;IoU\left(I_{16},I_{i}\right)\rangle ,\;\;\;\;i\leq 16$$

RCVs numerically summarize the proportional contribution of the body parts captured in a cropped image. We expect most of the IoU components of the vector will be 0, with only a few having values greater than 0 (as is the case for adjacent body parts). The component corresponding to the dominant body part will have the highest value. We found RCVs provide cues to the network that helps it better resolve overlap conflict when a concealed item is partially contained in the ROI. The cropped images pass through feature extraction blocks and the masks are downsampled to match the dimensions of the extracted features. We used the first five residual convolution blocks of MobileNetV2 CNN [43] (pretrained on ImageNet [44]) for feature extraction. The extracted features, downsampled masks, and RCVs are separated into *m* = 4 sub-sequences, each containing *p* = n/m contiguous temporal components that are fed to a dual pipeline, multi-phase sub-network. During each phase, the cropped body part image pipeline (*CBI-pipeline*, *blue path* in Figure 8) encodes textures of the entire cropped images, while the *ROI-pipeline* (*red path* in Figure 8) is designed to extract textures precisely from the ROIs in the cropped images. This is achieved by computing the element-wise multiplication of the residual convolution block output of the *CBI-pipeline* and the convoluted ROI masks. The resulting tensor is passed to the residual CNN block of the *ROI-pipeline*. The dual pipelines ensure the network can detect anomalies that partially appear on the boundary of the body part’s ROI and aids the network to decide whether to attribute the detected anomaly to the ROI. This is especially useful when a concealed object partially appears in the ROI but is fully captured in the cropped image. The output of the residual CNN block in the *ROI-pipeline* of the final phase and the RCVs are fed to a fully connected (FCN) block, which outputs the probability that a concealed item is present in one or more of the cropped images in the given sub-sequence. The final classification of a body part (given all cropped images) is aggregated as the max probability of the sub-sequences. To summarize, the proposed two-phase, dual pipeline CNN anomaly detector (RaadNet) is made up of nine residual CNN blocks (five in the feature extractor) and an FCN block. The MobileNet feature extractor has 12 2D convolution layers and our custom *Sub-Network* has 13 3D convolution layers.

#### Training and Inference with RaadNet Ensemble Classifiers

Our two-phase, dual pipeline network has about 2.47M parameters (5.19M flops). We trained three classifiers on overlapping, equal-sized subsets of the training set. This was executed with a *3-fold* stratified learning scheme where each classifier was trained on two subsets and validated on the other subset. Special care was taken to segment the training set into three subsets with similar distributions of threat object occurrence in each body part. Each classifier was trained for 80 epochs with a batch size of 64 using the Adam optimizer [45] and a dynamic learning rate starting at $1\times 10^{-3}$ and decreasing to $5\times 10^{-5}$ between the 9th and 72nd epoch by a non-linear cosine function. The weights of the MobileNet feature extractor were frozen for most of the training epochs and finetuned during the last five epochs. During training, we resampled sequences of cropped images with concealed items and augmented the images by moving the cropping window in a confined area of the ROI, zooming in and out, horizontally flipping images, and adjusting the image contrast.

At inference, backscatter images of a scan were first segmented into the different body parts. Cropped images of each body part (along with their ROI masks and derived RCVs) were fed to each of the three classifiers. We show the detection performance of a single classifier trained on two-thirds of the training data, and validated on the other one-third, in Section 4.2. The final anomaly detection prediction for a body part is aggregated as the mean threat object probability of the three ensemble classifiers. Concealed threat object detection is performed for each body part of a scan as described above. We report the log-loss performance of our proposed ensemble classifier on the TSA-PSD test set in Section 4.3.

### 3.3. Extension: Learning Local Optimal 2D Pose Correction

The multi-view coherent pose bundle adjustment algorithm proposed in Section 3.1.2 optimizes a global 3D pose by aggregating the 2D poses of a scan. Therefore, the resulting coherent 2D poses that are projected from the optimized 3D pose are consistent with the source 3D pose and only differ by their viewpoint transformation. Although 2D poses projected from a global optimum 3D pose are ideal, in practice, we observe that local optima 2D poses are more accurate, as a subject may move slightly when being scanned, thereby causing subtle but noticeable variations in 2D poses from frame to frame. Furthermore, while manually labeling a small subset of the TSA-PSD, we noticed that the preferred location of a keypoint on the body is rarely fixed, as the viewpoint changes and may move a few pixels in any direction depending on the orientation and positioning of the person in the frame. For example, the preferred location of the neck keypoint in a frontal view of a subject is at the center of the axis between the right and left shoulders. However, in the back view of the same subject, the preferred location of the neck keypoint moves a few pixels upward above the axis between the shoulders. This is because the chest region is anatomically lower than the upper back region. Hence, we hypothesize that individually optimizing the 2D pose in each frame while taking into account cues from the 2D poses in neighboring frames would be a better approach to correct sub-optimal 2D poses. Therefore, we explored another method for estimating coherent 2D poses that is learnable and more adaptable than the RANSAC-optimized multi-view pose bundle adjustment approach previously discussed (Section 3.1.2). Note, either method can be used interchangeably to refine estimated 2D poses and generate coherent 2D poses used to segment the body parts that are subsequently fed to RaadNet for anomaly detection.

#### 3.3.1. Weakly Supervised 3D to 2D Pose Correction Network

**Figure 9:**
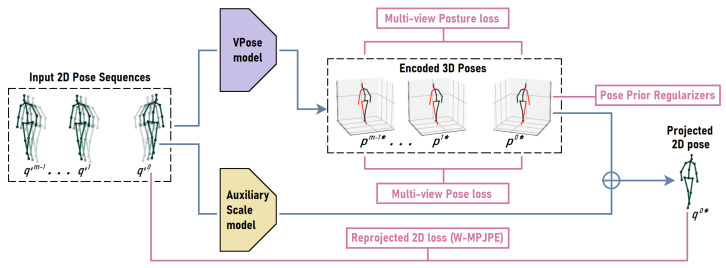
An illustration (with one training example) of our weakly supervised scheme for encoding a more correct 3D pose given sub-optimal 2D poses. Each training sample contains *m* types of 2D pose inputs. $q^{0}$ is **the primary** sequence of 2D poses for which we optimize the pose prior regularizers and reprojected 2D loss. $q^{i}$ (i≠0) are matching sequences of 2D poses of the same scan taken from other viewpoints. These 2D poses are fed to the weakly supervised pipeline to estimate their 3D poses ($p^{0*},p^{1*},\cdots p^{(m-1)*}$) and the 3D-to-2D scale factor of the primary sequence ($s^{0}$). The encoded 3D pose, $p^{0*}$, and the corresponding scale factor are combined to project a more correct 2D pose and minimize the reprojected 2D loss. The reprojected 2D pose is the output of the network. Pose prior regularizers [5] are also enforced on $p^{0*}$. In addition, we minimize the multi-view pose and posture losses between $p^{0*}$ and $p^{1*}\cdots p^{(m-1)*}$ [4]. This weakly supervised framework is used to retrain VPose [46] without 2D or 3D pose ground-truth annotations. In our experiments, we set m=4.

We propose an alternative approach that optimizes the 2D pose in each frame using a weakly supervised 3D pose estimator framework we proposed in previous works [4,5]. We have chosen the VideoPose3D pose estimator network (**VPose**) [46] pretrained on the Human3.6m dataset (**H36M**) [47] for this purpose. VPose is a temporal dilated convolutional neural network that takes as input a contiguous sequence of 2D poses (*x* and *y* keypoint locations) and outputs a 3D pose (*x*, *y*, and *z* joint locations). In our setup and configuration of the VPose architecture, given a sequence of *n* 2D poses, the network predicts the 3D pose corresponding to the median 2D pose in the sequence (i.e., 2D pose at index ⌊n/2⌋ in the sequence). We refer to this median frame, 2D, and 3D pose as the **active frame, 2D, and 3D pose** of the sequence (denoted by ‘*’). Note, *n* must be an odd number. We conduced the experiment with n∈{9,27}. When we retrained the VPose network with weak supervision on HRNet-estimated 2D poses of TSA-PSD, the 3D pose network adapted to the dataset and learned to encode more correct and coherent 3D poses given sub-optimal 2D poses. As in Section 3.1.2, we projected the encoded 3D pose to 2D image space to retrieve the corrected 2D pose. The weakly supervised framework illustrated in Figure 9 shows how VPose is retrained without ground-truth 3D pose annotations.

During training, the network receives a **primary sequence** of 2D poses and m−1**secondary sequences** of 2D poses. The secondary sequence of 2D poses matches the 2D poses of the primary sequence, except they are mostly taken from different viewpoints or frames. For example, take a **.a3daps** scan with 64 frames, m=3, and the size of the sequence n=27. The primary sequence can be 2D poses from frames 1–27 and the other two secondary sequences are 2D poses from frames 22–48 and 43–5. Multi-view consistency pose and posture loss are optimized between the network-encoded active 3D poses of the primary and secondary sequences. This ensures that 3D poses from different viewpoints are identical up to the viewpoint transformation. Pose prior regularization constraints are only enforced on the active 3D pose $p^{0*}$, forcing the network to estimate geometrically correct 3D poses. Additionally, a 2D reprojection loss is computed between the active projected 2D pose, $q^{0*}$, and the active 2D pose input in the primary sequence, $q^{{}^{\prime }0*}$. The reprojection loss completes the encoder–decoder nature of the framework and ensures that the 2D pose derived from the encoded 3D pose is somewhat similar to the input 2D pose. Note that VPose estimates 3D poses with respect to the root (pelvis) joint. That means that the pelvis of the encoded 3D poses is always at the Cartesian origin or close to it. We use an auxiliary pretrained scale model to estimate the 3D–2D pose scale factor. We discuss these loss terms in more detail below.

#### 3.3.2. Weakly Supervised Loss Terms

The weakly supervised loss terms that are minimized when training the proposed pose correction model are the reprojected 2D loss [46], multi-view pose consistency loss, and multi-view posture consistency loss [4]. We discuss these three loss terms as follows.

##### Reprojected 2D Loss (R2D)

The reproject 2D loss is defined in Equation (2) as the weighted mean per joint position error (W-MPJPE) that computes the Euclidean distance between the primary input 2D poses, $q^{\prime }$, in a batch and their corresponding orthographic projected 2D poses, *q*, weighted by the confidence score of the keypoint.

(2) $$\begin{array}{cc} q^{0*} & =\left(p_{\left[x,y\right]}^{0*}\cdot s^{0*}\right)+q_{r}^{{}^{\prime }0*}\\ L_{R2D} & =\frac{1}{\left|Q^{0*}\right|\left|J\right|}\sum_{i=0}^{\left|Q^{0*}\right|}\sum_{j=0}^{\left|J\right|}w_{i,j}\cdot {∥q_{i,j}-q_{i,j}^{\prime }∥}_{2} \end{array}$$

where $p_{\left[x,y\right]}^{0*}$ is the *x* and *y* components of the active encoded 3D pose of the primary sequence, $s^{0*}$ is the corresponding 3D–2D scale factor and $q_{r}^{{}^{\prime }0*}$ is the position of the root (pelvis) keypoint of the active 2D pose in the primary input sequence. $Q^{0*}$ is the set of active 2D poses of the primary 2D pose sequences in the training batch that the network estimates and projects. *J* is the set of keypoints of a 2D pose. $q_{i,j}$ is the 2D position of keypoint *j* of pose *i* in $Q^{0*}$ and $q_{i,j}^{\prime }$ is the position of the keypoint in the corresponding input 2D pose. $w_{i,j}$ is the confidence score of the keypoint taken from the confidence map output of the Deep-HRNet 2D pose detector used to generate the input 2D poses. A keypoint’s confidence score is a number between 0 and 1 that indicates how confident the 2D pose detector is that the keypoint position is correctly estimated. Typically, incorrectly estimated keypoints have lower confidence scores. Therefore, by weighting the reprojected 2D loss by the keypoints’ confidence score, we prevent the network from fitting to suboptimal keypoints, which are precisely the keypoints we want the network to correct.

##### Multi-View Pose Consistency Loss

We propose a multi-view pose consistency loss defined in Equation (3) as the mean Euclidean distance between pairs of joints of estimated 3D poses (at different frames) of the same scan after frame alignment. Note that we take the 3D pose of the first, full frontal view frame (i.e., the viewpoint at $0^{\circ }$) to be the **absolute 3D pose** of the scan.

(3) $$\begin{array}{cc} p^{f*} & =T(p^{f*},R^{f*})\\ L_{pose} & =\frac{1}{\left(m-1)|P||J\right|}\sum_{i\in P}\sum_{j\in J}\sum_{f=1}^{m}{∥p_{i,j}^{0*}-p_{i,j}^{f*}∥}_{2} \end{array}$$

where $R^{f*}$ is the rotation matrix used by the transformation function T to rotate the encoded 3D pose $p^{f*}$ of frame *f* to the absolute frame 3D pose $p^{f*}$. *P* is the number of training examples in a batch. A set of *m* multi-view 3D poses is encoded per training example. *m* is the preset number of multi-view 3D poses of the same scan estimated in a batch. *J* is the set of joints of a 3D pose. $p_{i,j}^{0*}$ and $p_{i,j}^{f*}$ are the encoded 3D poses of the primary sequence and a corresponding secondary sequence focused on a different viewpoint. Both 3D poses are rotated to the universal absolute frame. Multi-view pose consistency loss effectively optimizes the coherency between estimated 3D poses from different viewpoints without ground-truth 3D pose annotation.

##### Multi-View Posture Consistency Loss

Mult-view posture loss is similar to multi-view pose consistency in that it leverages multi-view information but differs by optimizing posture similarity. A 3D pose can be decoupled into 3D posture and the global orientation of the posture. The 3D posture captures the positioning of joints relative to each other and independent of the viewpoint. Hence, posture is invariant to the global orientation of the pose. Our weakly supervised multi-view posture consistency loss is defined in Equation (4) as the mean L1-norm between pairs of orientation-aligned unit vectors of bones of estimated 3D poses (at different frames) of the same scan.

(4) $$L_{posture}=\frac{1}{\left(m-1)|P||B\right|}\sum_{i\in P}\sum_{j\in B}\sum_{f=1}^{m}{∥\alpha_{j}{\hat{b}}_{i,j}^{f}-\alpha_{j}{\hat{b}}_{i,j}^{f-1}∥}_{1}$$

where *B* is the set of bones in a standard 3D pose skeletal structure. ${\hat{b}}_{i,j}^{f}$ is the unit vector of bone *j* of encoded 3D pose *i* of frame *f* after our bone orientation alignment procedure proposed in [4]. The novel bone orientation alignment procedure is a translation, rotation, and scale-invariant operation that transforms 3D poses in a standardized manner to retrieve the true orientation of a bone relative to other neighboring bones, irrespective of the global size, positioning, and orientation of a 3D pose. $\alpha_{j}$ is computed from the batch of estimated 3D poses as the mean bone length of bone *j*. This multi-view posture consistency loss term allows the weakly supervised model to finetune the accuracy of estimated 3D pose joints by focusing on posture precision.

#### 3.3.3. 3D Pose Prior Regularizers

3D pose regularization is a critical component of our proposed weakly supervised pose correction network. The idea is to enforce certain natural biomechanical and kinematic constraints of a 3D pose to nudge the network towards encoding highly probable and geometrically correct 3D poses. To this end, we maximize the log-likelihood of our bone proportion and joint mobility constraints proposed in [5].

##### Bone Proportion Constraint (BPC)

We propose a bone proportion regularization constraint that is formulated to enforce the realistic average human proportion between lengths of bones in a 3D pose. BPC is inspired by the Human System of Proportions [48,49,50] studied by physicians and artists, which establishes that the length of the human body parts are related and can be quantified in terms of the head or cranium length even though the bone structure may differ per individual. We deduce from this premise that human bone lengths are related by a standard proportion, irrespective of a person’s height or size. We encode these proportions in terms of bone length ratios between pairs of adjacent bones in a standard 3D pose skeletal structure. Data samples for each bone ratio pair are modeled by a Gaussian distribution parameterized by their mean, μ, and variance, $\sigma^{2}$, so that the bone proportion constraint defined in Equation (5) can maximize the mean log-likelihood of all bone proportion pairs.

(5) $$L_{BPC}=-\frac{1}{\left|P||B\right|}\sum_{i\in P}\sum_{j:(k,l)\in B}\ln\left(f_{\mu_{j},\sigma_{j}^{2}}\left(\frac{b_{i,k}}{b_{i,l}}\right)+1\right)$$

Before training, the bone proportion parameters are generated from annotated 3D poses of H36M or coherent TSA-PSD 3D poses from Section 3.1.2. During training, the mean, μ, and variance, $\sigma^{2}$, for bone ratios are used to compute the likelihood of the bone proportions of encoded 3D poses by the probability density function, $f_{\mu_{j},\sigma_{j}^{2}}$. Note $b_{i,k}$ and $b_{i,l}$ are adjacent bones of the active 3D pose encoded for the primary sequence. BPC ensures that the bone lengths of encoded 3D poses are not out of proportion.

##### Joint Mobility Constraint (JMC)

The joint mobility regularization constraint is designed to monitor the direction and degree of rotation of movable joints in a 3D pose and their associated bones, thereby nudging the network towards encoding 3D poses with plausible relative orientation between adjacent bones. Similar to BPC, JMC is modeled by a multivariate probability density function that evaluates the likelihood of a given bone orientation. The true orientation of each bone in a 3D pose is extracted after our novel bone orientation alignment, first introduced in [5] and discussed in Section 3.3.2. Joint mobility is encoded in terms of the aligned unit vector of an associated bone and parameterized by the per-axis mean (μ) and covariance matrix (Σ) of the set of unit vectors of the given bone. As in BPC, JMC parameters are generated from annotated 3D poses of H36M or coherent TSA-PSD 3D poses before training is resumed. During training, the joint mobility constraint defined in Equation (6) is enforced by maximizing the mean log-likelihood of bone orientations.

(6) $$L_{JMC}=-\frac{1}{\left|P||B\right|}\sum_{i\in P}\sum_{j\in B}\ln\left(f_{\mu_{j},\Sigma_{j}}\left({\hat{b}}_{i,j}\right)+1\right)$$

where $f_{\mu_{j},\Sigma_{j}}$ is the multivariate probability density function of the Gaussian distributed bone unit vectors. ${\hat{b}}_{i,j}$ is the orientation aligned unit vector of bone *j* of estimated 3D pose *i*. Note, similar to BPC, *P* refers to the set of active 3D poses encoded for the primary sequence. Observe how this formulation of the proposed joint mobility constraint influences the network to encode 3D poses with highly probable 3D postures because the likelihood of the relative orientation of each bone is maximized. This is especially effective for the TSA dataset because all scan subjects are expected to have a similar posture; standing upright with legs spread apart and hands raised above the head. This joint mobility constraint will explicitly bias the network to estimate 3D poses with the expected posture when the JMC parameters are derived from RANSAC-optimized coherent 3D poses of TSA-PSD.

## 4. Results

We conduct a series of experiments to validate our proposed method. First, we evaluated the correctness of our 2D pose refinement procedure (in Section 4.1) and the accuracy of our anomaly detection network (in Section 4.2). We then combined three states of our proposed anomaly detection network (each trained on a different subset of training data) into an ensemble classifier and compared the performance of our ensemble classifier to state-of-the-art algorithms applied to TSA-PSD in Section 4.3.

### 4.1. Evaluation of 2D Pose Refinement for MWS Images

We evaluated our proposed keypoint correction process to show the relevance of our approach that adapts an RGB pretrained pose encoder to estimate more accurate poses on backscatter MWS images without supervision. Table 1 shows the mean per joint position error (MPJPE) computed between predicted keypoint positions and manually labeled ground-truth positions. The final stage of our RANSAC-optimized pose refinement (*Coherent Pose-1*) decreases the error of estimated keypoints by 68%. We went on to show that this boost in accuracy is carried over to the anomaly detection network when trained with better segmented images. Note, however, that the consolidation of globally optimum coherent poses can sometimes come at the expense of local optima keypoint positions. We observe this consequence in the right and left hip and right knee keypoints where the coherent poses degrade the accuracy of the refined poses. This is because the pixel locations of these keypoints are particularly volatile from frame to frame as the viewpoint of the person changes. Our second 3D–2D pose correction model approach avoids this error because it optimizes the 2D pose in each viewpoint individually while taking cues from neighboring viewpoints. By so doing, it effectively decreases the error of HRNet estimated keypoints by 74% (*Coherent Pose-3*) when pose priors are generated from RANSAC-optimized 3D poses of TSA-PSD and 72% when the priors are generated from the H36M dataset with more pose variations.

### 4.2. Concealed Item Detection with RaadNet

We conducted ablation experiments on our anomaly detection network trained on different types of segmented body part images and varying inputs. We presented a comprehensive evaluation of our methods in comparison to published works on concealed item detection on the TSA dataset in Table 2. Our proposed method of using refined 2D keypoints to segment the human body parts consistently outperforms other published work on 2D concealed item detection in TSA-PSD in all metrics. Our RaadNet detector, trained on body part images segmented by coherent keypoints, ROI masks, and RCVs, performs at an average F1 Score of 98.6% on a disjoint validation set and 0.0751 log-loss on the test set. The log-loss, ϵ, on the test set of TSA-PSD is defined in Equation (7) between predicted threat probabilities, $\hat{y}$, and the ground-truth binary label, *y*, of all *N* = 17×1338 body parts and scan subjects in the test set. “ln” is natural logarithm.

(7) $$\epsilon =-\frac{1}{N}\sum_{i=1}^{N}\left[y_{i}\ln\left({\hat{y}}_{i}\right)+\left(1-y_{i}\right)\ln\left(1-{\hat{y}}_{i}\right)\right]$$

By performing more precise body part segmentation on MWS images using refined 2D keypoints, we improved our network’s ability to accurately detect concealed items by 53% (0.0751 test set log-loss), outperforming the published state-of-the-art method [19] (at 0.0913 test log-loss) by 17%. This is further extended to a 38% decrease in log-loss by our three-ensemble classifiers. Note that the methods in the top three rows of Table 2 are not directly comparable to our results because those works detect anomalies in a small subset of body parts (e.g., chest, thigh, arm, and back), whereas our method detects concealed items on all body parts and the reported values reflect the cumulative performance on all body parts. To aid comparison with previous works, we show their best results reported for a single body part. Our ablation study highlights the importance of RCVs and ROI masks. As expected, the use of masks improves the confidence of classification in *Abl-2* (decrease in log-loss). Although, this is at the expense of classification accuracy, which is recovered by supplying RCVs. This is because ROI masks may exclude parts of objects on the boundary of body parts, whereas RCVs inform the network how much of the objects are contained in the ROI, hence allowing the network to make a better decision of attributing detected concealed objects to body parts.

### 4.3. Comparison to TSA-PSD Proprietary Classifiers

**Figure 10:**
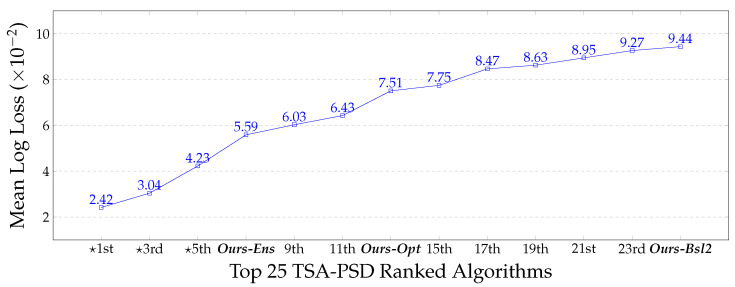
Top-ranked TSA-PSD algorithms on the Kaggle Leaderboard. (★) indicates algorithms reported to have used 3D image files. Our ensemble RaadNet ranks 7th, placing in the proprietary category. This makes our method the only published, comprehensive work that ranks in the top eight algorithms for anomaly detection on human body parts in the TSA dataset.

The anomaly detection accuracy of RaadNet is further improved with three-ensemble classifiers (*Ours-Ens*), achieving up to 0.0559 mean log-loss on the test set (ranked 7th in Figure 10). Note that the mean log-loss is the only evaluation metric reported for the test set because the ground-truth labels are private to TSA. This makes our proposed method the only comprehensive, fully disclosed work that places in the top eight proprietary categories on the TSA Leaderboard. Details of the top 11 algorithms are proprietary and undisclosed to the public. Most of the top eight methods are reported to use a combination of 3D volumetric data (*330 MB* per file) and 2D (*10–41 MB*) image data (1st), whereas we use only the smallest 2D image data available (*10 MB, **.aps** files*). RaadNet may be directly compared to the 6th-ranking method, which uses 2D data and multiple classifiers specialized for each body part. In contrast, we use only three-ensemble classifiers, with each component classifier trained on a disjoint subset of all body parts. We observed that all our baseline classifiers have a higher rate of false negatives than false positives. In other words, RaadNet is more likely to miss a concealed item than to generate false alarms. The difference narrows and the false negative rate decreases as more precise body part segmentation is used in *Ours-Ens* and *Ours-Opt*, highlighting the importance of accurate body segmentation in backscatter MWS images.

## 5. Conclusions

We have shown how improved 2D human pose estimation and the consequent improvement in body part segmentation can lead to a significant performance boost in the task of body part anomaly detection. Adapting 2D pose detectors trained on RGB images to estimate the keypoints of people in backscatter MWS images is non-trivial without ground-truth annotations but, as we show, can be very rewarding when executed well. Our proposed keypoint refinement procedure and unsupervised body part segmentation algorithm described in Section 3.1 enables us to correct sub-optimal 2D poses and accurately segment the body parts of people in MWS images. Subsequently, this allows us to train our proposed body-part-aware anomaly detection network on cropped images of segmented human body parts in MWS backscatter images. Given the precise segmentation of body parts, we design an effective body-part-aware CNN that can be trained with minimal (binary labels) annotation to detect concealed threat objects on all body parts in tandem. Our proposed approach achieves SOTA detection accuracy and ranks among the proprietary (top eight) algorithms evaluated on the TSA passenger screening dataset.

## Figures and Tables

**Table 1:** Accuracy of 2D poses derived by the naive keypoint selection (*Generic Pose*) and our proposed method for guided keypoint selection (*Confined Pose* of Section 3.1.1). Coherent pose refers to poses optimized by RANSAC bundle adjustment (*Coherent Pose-1* of Section 3.1.2) and 3D–2D pose correction model trained with H36M generated pose priors (*Coherent Pose-2*) and TSA-PSD generated pose priors (*Coherent Pose-3*) in Section 3.3. The errors are computed as the mean L2-norm between manually labeled keypoint locations and estimated keypoint locations of 50 scans (800 images). *R.Sh* refers to the right shoulder, *L.Ke* left knee and so on. *Avg.* is the mean of all keypoints. Best in bold, second best is underlined.

mm	R.Sh	R.Eb	R.Wr	L.Sh	L.Eb	L.Wr	R.Hp	R.Ke	R.Ak	L.Hp	L.Ke	L.Ak	*Avg.*
*Generic Pose*	115.7	191.5	119.1	100.4	173.4	117.6	88.49	115.3	137.3	79.91	105.8	134.5	123.2
*Confined Pose*	55.2	36.9	35.2	52.3	44.4	42.6	65.1	38.5	42.9	61.6	36.9	43.6	46.3
*Coherent Pose-1*	40.2	32.5	19.9	38.1	31.4	23.6	82.8	38.9	22.4	78.1	35.8	21.0	38.7
*Coherent Pose-2*	37.4	30.2	19.5	35.8	31.5	20.2	64.5	38.3	21.1	61.2	35.3	**20.4**	34.6
*Coherent Pose-3*	**35.6**	**28.0**	**17.1**	**35.2**	**17.5**	**17.3**	**61.8**	**36.0**	**20.2**	**61.2**	**35.1**	20.6	**32.1**

**Table 2:** Comparison of RaadNet and our proposed body segmentation to other published work on TSA-PSD. *Bsl-1* is our baseline network trained with fixed ROI segmented body parts with ROI masks and RCVs. *Bsl-2* is our baseline RaadNet trained with images segmented using original Deep-HRNet keypoints (without refinement), masks, and RCVs. *Abl-1*, *Abl-2*, and *Opt.* are our networks trained with body parts segmented using refined and coherent keypoints (Section 3.1.2). *Abl-1* is without ROI masks and RCVs, *Abl-2* is without RCVs, and *Opt.* is with all three inputs, i.e., cropped images of body parts, ROI masks, and RCVs. Best in bold, second best is underlined. The arrows in header row indicate whether larger or smaller numbers are better for a given metric.

Body Part Anomaly Detection Methods	Validation Set						Test
	Avg.F1 ↑	F1.Score ↑	Precision ↑	Recall ↑	Accuracy ↑	Logloss ↓	Logloss ↓
FastNet (*) [14]	0.8890	-	-	-	-	-	-
AlexNet-1 (*) [18]	-	-	-	-	-	**0.0088**	-
AlexNet-2 (*) [19]	0.9828	-	-	-	-	-	0.0913
RaadNet + FixedSeg. [18] + Mask + RCV (*Bsl-1*)	0.9761	0.9555	0.9628	0.9487	0.9723	0.0201	0.1384
RaadNet + Unref.PoseSeg. + Mask + RCV (*Bsl-2*)	0.9184	0.7526	0.8652	0.6659	0.9108	0.1282	0.1608
*Ours* RaadNet + Co.PoseSeg. (*Abl-1*)	0.9775	0.9581	0.9655	0.9505	0.9766	0.0143	0.0934
*Ours* RaadNet + Co.PoseSeg. + Mask (*Abl-2*)	0.9637	0.9540	0.9550	0.9531	0.9687	0.0131	0.0886
*Ours* RaadNet + Co.PoseSeg. + Mask + RCV (*Opt.*)	**0.9859**	**0.9738**	**0.9941**	**0.9544**	**0.9946**	0.0097	**0.0751**

## Data Availability

The TSA Passenger Screening Dataset used in this study can be obtained from the Kaggle challenge website https://www.kaggle.com/competitions/passenger-screening-algorithm-challenge/data (accessed on 3 November 2018). The Human3.6M dataset also used in this study can be obtained at the official website http://vision.imar.ro/human3.6m/description.php (accessed on 20 February 2019).

## Abbreviations

The following abbreviations are used in this manuscript:

HIPAA	Health Insurance Portability and Accountability Act
TSA	Transport Security Administration
TSA-PSD	Transport Security Administration Passenger Screening Challenge Dataset
HD-AIT	High Definition-Advanced Imaging Technology system
MWS	Millimeter Wave Scan images
H36M	Human3.6m 3D pose dataset
SOTA	State of the Art
ROI	Region of Interest
RCV	Region Composite Vector
CNN	Convolutional Neural Network
FCN	Fully Connected Network
CBI	Cropped Body part Image
GT	Ground Truth
RaadNet	ROI Attentive Anomaly Detection Network
W-MPJPE	Weighted Mean Per Joint Position Error
VPose	VideoPose3D Estimator Network
ICPR	International Conference on Pattern Recognition
NSF	National Science Foundation
